# Nanohydrogels: Advanced Polymeric Nanomaterials in the Era of Nanotechnology for Robust Functionalization and Cumulative Applications

**DOI:** 10.3390/ijms23041943

**Published:** 2022-02-09

**Authors:** Mohzibudin Z. Quazi, Nokyoung Park

**Affiliations:** Department of Chemistry, The Natural Science Research Institute, Myongji University, 116 Myongji ro, Yongin 17058, Gyeonggi-do, Korea; mhzb1195@gmail.com

**Keywords:** nanohydrogels, biodegradable, drug delivery, cancer therapy, crosslinked polymer network, stimuli-responsive, gene therapy, hybrid nanohydrogel

## Abstract

In the era of nanotechnology, the synthesis of nanomaterials for advanced applications has grown enormously. Effective therapeutics and functionalization of effective drugs using nano-vehicles are considered highly productive and selectively necessary. Polymeric nanomaterials have shown their impact and influential role in this process. Polymeric nanomaterials in molecular science are well facilitated due to their low cytotoxic behavior, robust functionalization, and practical approach towards in vitro and in vivo therapeutics. This review highlights a brief discussion on recent techniques used in nanohydrogel designs, biomedical applications, and the applied role of nanohydrogels in the construction of advanced therapeutics. We reviewed recent studies on nanohydrogels for their wide applications in building strategies for advantageously controlled biological applications. The classification of polymers is based on their sources of origin. Nanohydrogel studies are based on their polymeric types and their endorsed utilization for reported applications. Nanotechnology has developed significantly in the past decades. The novel and active role of nano biomaterials with amplified aspects are consistently being studied to minimize the deleterious practices and side effects. Here, we put forth challenges and discuss the outlook regarding the role of nanohydrogels, with future perspectives on delivering constructive strategies and overcoming the critical objectives in nanotherapeutic systems.

## 1. Introduction

Nanotechnology has been emerging as a field with high applicability at the nanosize scale, which has driven the concerns of researchers concerning the designing of nanocomposites, nanomaterial, nanocomplex, and nanoscale- based drug carriers [1,2,3]. Regardless of these extensive contributions and progressive approaches concerning therapeutic deliveries, numerous difficulties still exist [4,5,6]. These include designing suitable carriers/platforms for different purposes in the therapeutic distribution of drugs. Delivery of genetic materials, nucleic acids, encapsulated nanocomplex delivery onto specific sites to target damaged parts of the cells/body are also the major concerns [7]. The major cause associated with the low performance of carriers may be due to the interference of biological substances causing the disturbance. Similarly, plasma carrier instability, cytotoxicity, and early renal excretions are problematic. Nanotechnology has developed several nanotherapeutic systems [8], such as nanotubes [9], micelles [10], dendrimers [11], and hydroxyapatite NPs [12], for drug delivery purposes with advanced approaches. Nevertheless, studies have shown insufficient biodistribution, lacking biodegradability, cytotoxicity, and unstable performances in in vivo treatments that resulted in hindrance to the successful utilization of nanotherapeutic systems in clinical trials [13]. However, the clinical applications of most nanomedicines are impeded by the lack of specific localization of the therapeutic agents at the desired sites [14,15]. An ideal nanotherapeutic system must deliver therapeutic agents to sites of interest effectively as well as managing to overcome the side effects. Furthermore, studies have proposed that delivery systems must carry therapeutics through the vasculature without any leakage for the development of a prominent platform for drug delivery systems [16]. Nanotherapeutic systems must have selective accumulation on diseased cells or tissues and exert their therapeutic effect rather than accumulate on normal areas, which may lead to side effects. Nanotechnology involves nanomaterials with certain credentials, and polymeric nanomaterials have contributed at a large scale in nanotechnology. As nanomaterials have similar properties, there are considerable nanomaterials that have been employed in different roles in molecular science [17]. Nanomaterials are comprised of nanoparticles, nanocomposites, a mixture of protein-peptides and nanoparticles [18]. Nanomaterials have been employed as MOF-incorporated templates, polymeric origamis, and external triggered applications [19]. In the process of constructing nanomaterials with applied roles and potentially encountering the aforementioned issues, researchers have devised strategies of interlinked three dimensional polymer networks called hydrogels. Hydrogels are comprised of polymeric chain interactions. In the interim, hydrogels have received considerable attention as a novel approach of polymeric materials, particularly as biomaterials, showing great potential with a wide range of promising applications [20,21]. Hydrogels are constantly being developed, and presently researchers are involved in introducing nanohydrogels/nanogels on a large scale for various medical applications [22,23]. Nanohydrogels have been engineered with several natural, synthetic and hybrid polymers. Their potential application extends to such areas as in anticancer drugs, regenerative medicine, controlled drug delivery systems, immobilization matrix for biosensing elements, and tissue engineering due to their high resemblance to healthy tissues [21]. Biocompatibility and biostability with controlled pharmacokinetics and pharmacodynamics are the other most relevant parameters in in vivo functionalization due to the properties of the materials. Moreover, nanohydrogels have less surface to volume ratio, which facilitates movement through the cellular membrane and makes them more efficient at cell uptake than other standard nano cargos such as liposomes, micelles, nanoparticles and nanotubes [24,25]. Polymeric nanomaterials as building blocks for nanohydrogels, which are highly biocompatible and biodegradable, has been discovered in nanotechnology stream [26]. This is because of the monomer building blocks, which are safe for bioimmune compatibility and have significant inflammatory response in the microenvironments. Consequently, hydrogelators are used in building polymeric nanomaterials screened to biosafety. Nanohydrogels have been emerging to have a dual role as a hydrogel with nano particulate properties. Nanohydrogels are advantageous in hydrophilic and flexible roles, being versatile, high water uptake and prolong holding capacity.

The flexible and resourceful strategies used in the construction of the use of nanohydrogels have received significant attention in nanotechnology. Several considerable review papers have been published which have discussed synthesis and characterization, and the applications of biodegradable nanogels/nanohydrogels as drug carriers and nucleic acid carriers [27,28,29,30,31,32,33]. In this review article, we have comprehensively reviewed the earlier reported studies and strategies applied in the engineering of versatile polymeric networks used at the nano level in the construction of nanohydrogels. We have summarized the detailed information about synthesis, modulation, innovative changes, and intense research efforts made in the past and recent years on nanohydrogels. The role of employing nanohydrogels as a successful probe in biomedical applications, environmental protection, intelligent devices, cell cultures, protein productions, mechanical properties, and future perspectives are discussed.

## 2. Nanohydrogels

Hydrogels are three-dimensional (3D) polymeric networks that can hold large amounts of water but will not dissolve in the aqueous environment due to fundamental crosslinks in their structures. Hydrogels were studied and modified at nano levels to deliver the potential use of hydrogels and to reach out to multiple applications at once. Nanohydrogels have shown more advantages than earlier reported materials that created nanosized polymers and nanohydrogels and showed potential flexibility, versatile behavior, and high biocompatibility [33,34]. Generally, nanohydrogel’s classifications are based on their preparation, physical properties, source, ionic charges, biodegradability, and crosslinking. Based on these polymeric classifications, several polymers were earlier reported as natural source-based polymers. Natural polymers were again sub-classified in the manner of building blocks, construction strategies, microenvironmental response, and multiple chain reactions. Recently, researchers have found multiple sources of natural polymers in the engineering of nanohydrogels, whereas deoxyribonucleic acid (DNA) and other natural based polymers were used in earlier studies for constructing nanohydrogels [35,36,37]. Natural-based polymers have been widely studied for their high biodistributions, non-cytotoxic nature, biodegradable suitability, and ease in excretion or renal clearance [38]. Moreover, studies have shown the natural polymer’s uncontrolled structure, degradability, and drug release behavior could be altered by constructing synthetic and hybrid polymeric crosslinked network [39] s. Furthermore, synthetic polymers and hybrid polymers were used as hydrogelators or monomers for building nanohydrogels, which consist of polyamides, polyethylene glycol, polypeptides, polyesters, and poly-phosphazenes [40]. Synthetic polymers have emerged with high stability, controlled structures, and well-behaved drug release properties. However, synthetic polymers lack immunological concerns to natural extracellular matrix proteins [41,42]. Given the complexity and advantage of synthetic polymers, several studies were carried out by incorporating bioactive probes with synthetic polymers, as well as combinations of natural and synthetic polymers. Similarly, treating homopolymers with heteropolymers to establish a hybrid form of polymers which helps to reduce the earlier reported complexities with the advantageous approach [43,44]. In this part of the review, we will briefly discuss the fabrication of nanohydrogels based on their types of polymers and their successful labor in versatile fields [45]. Here, we have classified the fabrication methodologies and their roles based on the source of nanohydrogels and engineered strategies.

## 3. Classification of Nanohydrogels Based on Natural and Synthetic Polymers

**Figure 1:**
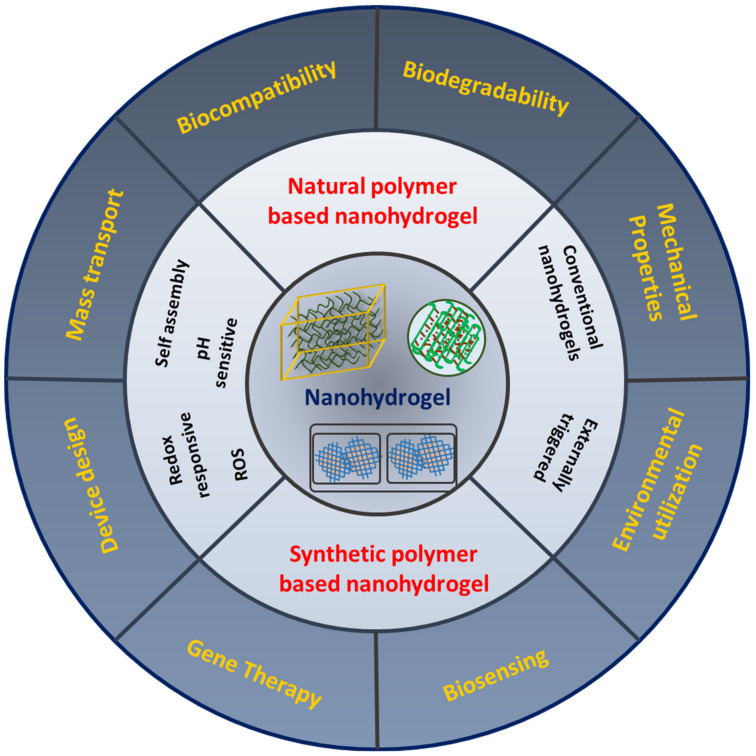
Schematic representation of nanohydrogel engineering based on the source of origin and applied functional role of nanohydrogels in nanotechnology.

Nanohydrogel classifications are based on different subjects, as explained earlier. Generally, based on the source and origin, nanohydrogels were classified into two major parts i.e., natural and synthetic polymeric network based nanohydrogels. Natural and synthetic polymer based nanohydrogels’ synthesis is feasible by using various approaches such as chemical cross-linking, ionic cross-linking, self-assembly, electrostatic interactions, reverse miniemuslions, hydrophobic interactions, and micelle cross-linking, whereas synthetic polymer-based nanohydrogels possibly use a similar mechanism to synthesize unique and more efficient nanohydrogels. Nanohydrogel classification and applications are being variably studied. A change in chemical composition, or a change in synthesis route, crosslinking designs such as the cross-linking method, cross-linking agents, or schematic methodologies could result in novel and unique nanohydrogels/biomaterials (Figure 1). Hybrid nanohydrogels are the recently trending subjects on which intrinsic studies stand operational. The formation of nanoparticle composite nanohydrogels in drug delivery, photothermal therapy, photodynamic therapies, and theranostic approaches is frequently studied.

### 3.1. Natural Polymers

Nanohydrogels engineered from natural sources have drawn remarkable attention due to their vast applications in pharmacy, agriculture, medicine, tissue engineering, cancer therapy, and drug delivery. Natural source isolated polymers such as gelatin, chondroitin, alginate, pullulan, etc. are known as natural polymers. These polymer-based engineered nanohydrogels were constructed with several different strategies such as ionic interaction-based nanohydrogels, crosslinking based nanohydrogels, etc. Here, we have classified natural source-based polymeric nanohydrogels and their constructive assemblies and functionalizations.

#### 3.1.1. Gelatin-Based Nanohydrogels

**Figure 2:**
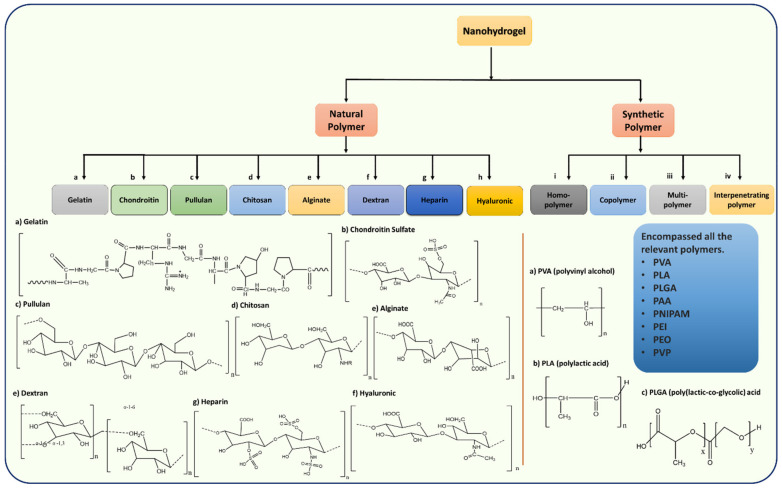
Schematic representation of the classification of nanohydrogels based on source and chemical structures of nanohydrogel’s natural and synthetic-based polymers.

Gelatin is produced by partial hydrolysis of collagens, and is a collection of peptides and proteins that are extracted from the skin, bones, and connective tissues. Collagen is obtained from the natural triple-helix structured collagen polymers by denaturation or hydrolysis of collagen polymers. Gelatin has a water absorbency of approximately 5–10 times its weight [46,47]. Furthermore, the commercial scale use of gelatin and accessibility made gelatin a great source of interest to synthesize nanohydrogels. Mimi et al. presented the polyethylene amine-based nanohydrogels with a biodegradable gelatin core to deliver siRNA in the cytoplasm of HeLa cells. This nanohydrogel was prepared by a physical crosslinking method with the size of nanohydrogel having an average diameter of 200 nm and +40 mV zeta potential. Using confocal laser scanning microscopic images, they have proved the intracellular uptake of siRNA into the cytoplasm of HeLa cells. The authors have concluded that the gelatin nanohydrogel shows low cytotoxicity as well as protects siRNA against enzymatic degradation and enhances cellular uptake by up to 84% [48]. Similarly, Jatariu et al. have synthesized ionic and covalent crosslinking-based chitosan-gelatin nanoparticles in reverse emulsion. Their gelatin-based nanoparticles have shown great swelling capacity and good hydrophilicity, which resulted in a slower release of loaded chloramphenicol by a diffusion mechanism up to 24 h [49]. Gelatin-based nanohydrogels were further studied for different applications in the delivery of cisplatin, rifampicin, and siRNA [46,50,51]. To further improve their stability, gelatin-based nanohydrogels are coated with PMAA to form gelatin PMAA nanohydrogels by coacervation/desolvation using formaldehyde as cross-linkers, followed by the radical polymerization of methacrylic acid. The rhodamine b encapsulated nanohydrogels display a sustained drug release profile for two weeks [52]. Gelatin-based polymers are further hybridized at a high scale with synthetic polymers, nanomaterials, and nanocomposites to enhance the multifunctionality of natural based polymers [53]. The applied utilization and modification of emerging polymeric networks gained more attention with alternative natural polymers (Figure 2). 

#### 3.1.2. Chondroitin-Based Nanohydrogel

**Figure 3:**
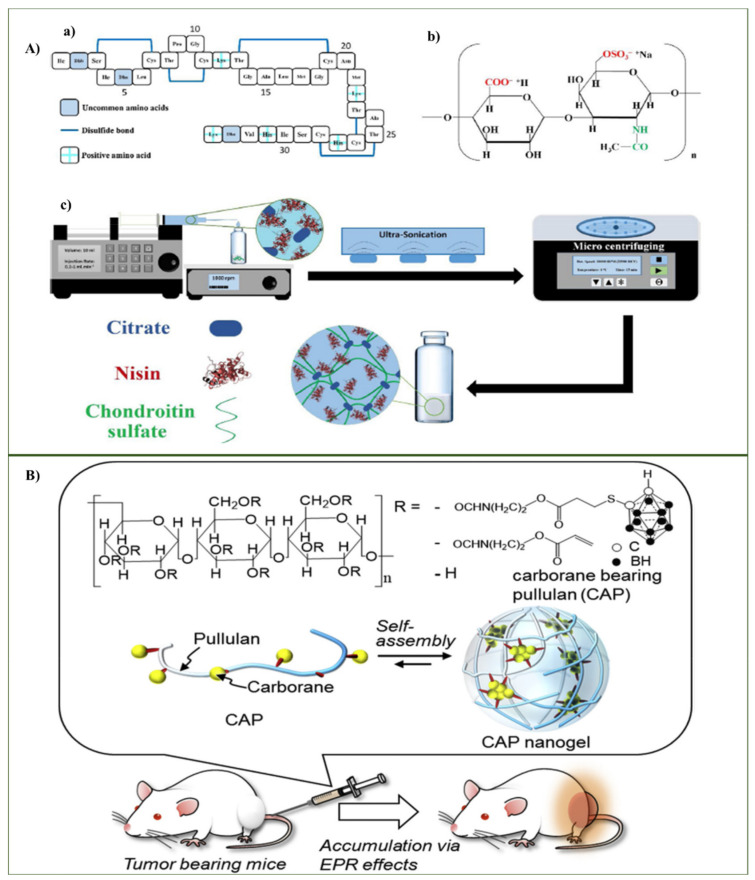
(**A**) (**a**) Nisin sequence (**b**) chemical structure of a Chondroitin sulfate unit (**c**) schematic of Chondroitin sulfate -nisin nanogel preparation. (Reprinted with permission from ref. [58] Copyright Elsevier 2016) (**B**) Chemical structure of carborane bearing pullulan for boron neutron capture therapy. (Reprinted with permission from ref. [60] Copyright Elsevier 2016).

Chondroitin is a sulfated glycosaminoglycan comprising D-glucuronic residue and N-Acetyl D-galactosamine residue. Chondroitin is referred to as chondroitin sulfate, which is usually isolated from the cartilage of animals [54]. Despite its greater variability of the source of extraction, the activity profile of chondroitin sulfate was observed because of its biodegradable and biocompatible nature. Chondroitin sulfate’s role has been studied as a primary choice to treat osteoarthritis and tissue engineering applications [55,56]. Chondroitin sulfate has also been used as a controlled release drug carrier and for anticancer drug targeting and protein delivery [57]. Chondroitin-based nanohydrogels have been emerged significantly in constructive practices such as copolymer assembly or different natural polymer assemblies to study chondroitin sulfate’s role in delivering effective therapeutics. Mohtashamian et al. have been studying the effective role of chondroitin sulfate-based nanohydrogels for their antibacterial and anticancer effects, in addition to their antimicrobial effects with controlled release using chondroitin sulfate-based nanohydrogels (Figure 3A). A Chondroitin sulphate-nisin nanohydrogel was synthesized using electrostatic self-assembly. The chondroitin anionic chains entrapped the cationic nisin. The study has been performed with quality by design approach [58]. In another study, Setayesh et al. developed chondroitin nanohydrogel using octadecyl amine grafted on chondroitin sulfate to form a self-assemble nanohydrogel. The reaction mechanism was executed using an amidation reaction between chondroitin and octadecyl amine [54]. The intracellular environment might inhibit the performance of nanostructures. Utilizing such obstacles in effective roles is a popular area in the research. Referring to such issues, Cao et al. have reported nanohydrogel construction through tethering zwitterionic nanohydrogels on a carboxylated silica work frame. Chondroitin sulfate mediated salt responsive platform fabricated via two step amidation reaction. The working mechanism involves an anti-polyelectrolyte effect of zwitterionic nanohydrogels combined with the recognition that chondroitin sulfate increases the adsorption efficiency that results in selectively fishing for low density lipoprotein (LDL) [59].

#### 3.1.3. Pullulan-Based Nanohydrogel

Pullulan-based nanohydrogels have a difference in charge with neutralized linear polysaccharides [61]. Pullulan is obtained from the starch of the fungus *Aureobasidium* and the fermentation process like black yeast. Pullulan is a polysaccharide polymer consisting of maltotriose units, also known as α-1,4; α-1,6 glucan. Three glucose units in maltotriose are connected by an α-1,4 glycosidic bond, whereas consecutive maltotriose units are connected by an α-1,6 glycosidic bond [62]. Pullulan has been studied widely due to the changes in functional derivatives that force pullulan to change its properties, which results in applied modifications [63]. The pullulan polymer is modified by hydrophobes such as cholesterol, which helps it to behave as amphiphilic molecules that could act as excellent nanohydrogel carriers with amphiphilic properties [64]. Pullulan-based nanohydrogels were actively used in vitro and in vivo. The modernization of nanohydrogels and the emergence of new alternative therapeutics has been observed, as in the “neutron capture therapy” of cancers. The delivery and accumulation of boron in the tumor, followed by radiation therapy, causes the neutrons to react with the boron to kill the tumor cells without harming the normal cells. Kawasaki et al. described pullulan-based nanohydrogel utilization as safe with regard to its delivering of carborane in in vivo treatments. Pullulan-based nanohydrogels produced no apparent cytotoxicity, although carboranes are known for their cytotoxicity. The pullulan nanohydrogel successfully minimized cytotoxicity of carborane and acted as dual role delivery and therapeutics in cancer cells and was successfully employed in boron neutron capture therapy (Figure 3B) [60].

#### 3.1.4. Chitosan-Based Nanohydrogel

Polymers have emerged as the material of choice for use in a wide range of medical and pharmaceutical applications, including the fabrication/coating of biomedical devices, therapeutic delivery systems, and tissue engineering [30,65]. Chitosan-based hydrogels are comprised of β-(1,4)-linked D-glucosamine and N-acetyl-D-glucosamine units. Chitosan-based hydrogels have been reported in earlier studies. The chitosan polymer has been known for its biocompatibility and biodistribution. Chitosan polymer-based nanohydrogels and modified chitosan hydrogels using functional materials and nanoparticles were reported for their biocompatibility and stimuli-responsive high efficacy in chemotherapeutics [66]. Chitosan is a cationic linear polysaccharide and hydrophilic in nature. Earlier, to enhance the efficacy of hydrophilic polymers, hydrophobic moieties or polymers were grafted in order to produce self-assembly behavior due to its amphiphilic nature. The amphiphilic interactions play a major role in the self-assembled engineering of nanohydrogels. Luckanagul et al. reported chitosan grafted pNIPAM copolymeric based nanohydrogels for thermoresponsive delivery of curcumin in cancer cells, which has shown effective and dose-dependent cytotoxicity against cancer cells [67]. Grafted polymers/copolymers based hydrogels have been studied for their change in intrinsic properties after their assembly with pNIPAM and other moieties [68]. Chitosan-based nanohydrogels were effectively utilized for their high biodistributions. These nanohydrogels were modified with ligands and receptors that are highly expressed in cancer cells to target specifically diseased areas [69,70].

#### 3.1.5. Alginate-Based Nanohydrogel

Alginate-based nanohydrogels are another well studied natural polymer derived nanohydrogel because of their biomedical applications. Alginates are the anionic polysaccharide, and consist of 1,4-linked β-D-mannuronic acid and α-L-guluronic acid. Alginate-based hydrogels were earlier reported as small drugs, encapsulation of protein, and delivery [71,72]. Chopra et al. reported the development of alginate-based nanohydrogels with gum acacia copolymer for its effective and wide range of applications in antibacterial activity [73]. Moreover, alginate-based nanohydrogels, which are nanomaterials composed of gels, were noted for their antibacterial and antimicrobial as well as regenerative medicinal applications [74,75]. Alginate had played a major role as a prodrug, and it has been studied earlier for its key role in biomedical applications due to the non-antigenicity, non-cytotoxicity, biocompatibility, and pH sensitivity [76] it possesses. Recently, several studies have been performed to explore the synergistic effects of alginate-based nanohydrogels in nanotherapeutics. Advance modification of nanohydrogels with low cytotoxicity and simultaneous detection of diseased cells are another growing concern in recent studies [77]. Podgorna et al. designed gadolinium-alginate nanohydrogels by a physical crosslinking and reverse emulsion technique to deliver hydrophilic drugs and analyzed them using magnetic resonance imaging. As they demonstrated, nanohydrogels with a hydrodynamic size of 110 nm can be employed as nano vehicles for theranostic applications [78]. Similarly, to explore the versatile application of alginate based nanohydrogels, Pei et al. prepared stimuli-driven and turn on theranostic prodrug nanohydrogel for cancer detection and therapy. Overexpression of folate receptors on the cancer cells and folic acid designed nanohydrogel achieved the folate receptor mediated targeting and pH/reduction dual responsive intracellular triggered release of the drug. The author has designed nanohydrogels by crosslinking the folate-terminated poly(ethylene glycol) and rhodamine b-terminated poly(ethylene glycol) modified oxidized alginate with cystamine. This was followed by the covalent conjugation of doxorubicin via an acid-labile Schiff based bond. The study shows that cancer associated, stimuli-driven and turn on theranostic prodrug nanohydrogels were designed for diagnosis and therapeutic purposes [79].

#### 3.1.6. Dextran-Based Nanohydrogel

Dextran polymers have emerged as a widely used polymer in several fields. Dextran-based nanohydrogels were reported for their sustained zinc release in antimicrobial applications [80]. Dextran-based nanohydrogels were earlier reported by Zhou et al. for efficient imaging of nanoprobes for adipose-derived stem cells. The construction of dextran-based nanohydrogel with a random diameter ranging in between 165 to 241 nm and with −10 to 10 mV surface potential was fabricated in three steps. It involves a three-phase procedure that involves the fabrication of dextran-based nanohydrogels, followed by the conjugation of fluorescent molecules with the nanohydrogels, and lastly the surface modification of the fluorescent nanohydrogels [81]. Dextran has been extensively studied for its copolymer/biopolymer role in modifying the intrinsic physical properties of other polymers, as well as dextran polymer, due to its neutral polysaccharide nature [27].

#### 3.1.7. Heparin-Based Nanohydrogel

Heparin is generally used as an anticoagulant that prevents the formation of blood clots. Heparin is used to treat and prevent blood clots caused by medical conditions. Heparin is a linear polysaccharide that consists of one to four linked disaccharides, random repeating units of uronic acid, and glucosamine residues. Heparin-based hydrogels have been studied for their versatile applicability and functionalization, including implantation, tissue engineering, biosensors, and drug-controlled release [82,83]. Heparin is composed of pyranosyluronic acid and glucosamine residues. Heparin nanohydrogels are anionic in nature, and Heparin based nanohydrogels have been used for drug delivery due to their crosslinking nature [84]. Heparin polymers have also been reported to have anticancer properties in earlier studies [85].

#### 3.1.8. Hyaluronic-Based Nanohydrogel

**Figure 4:**
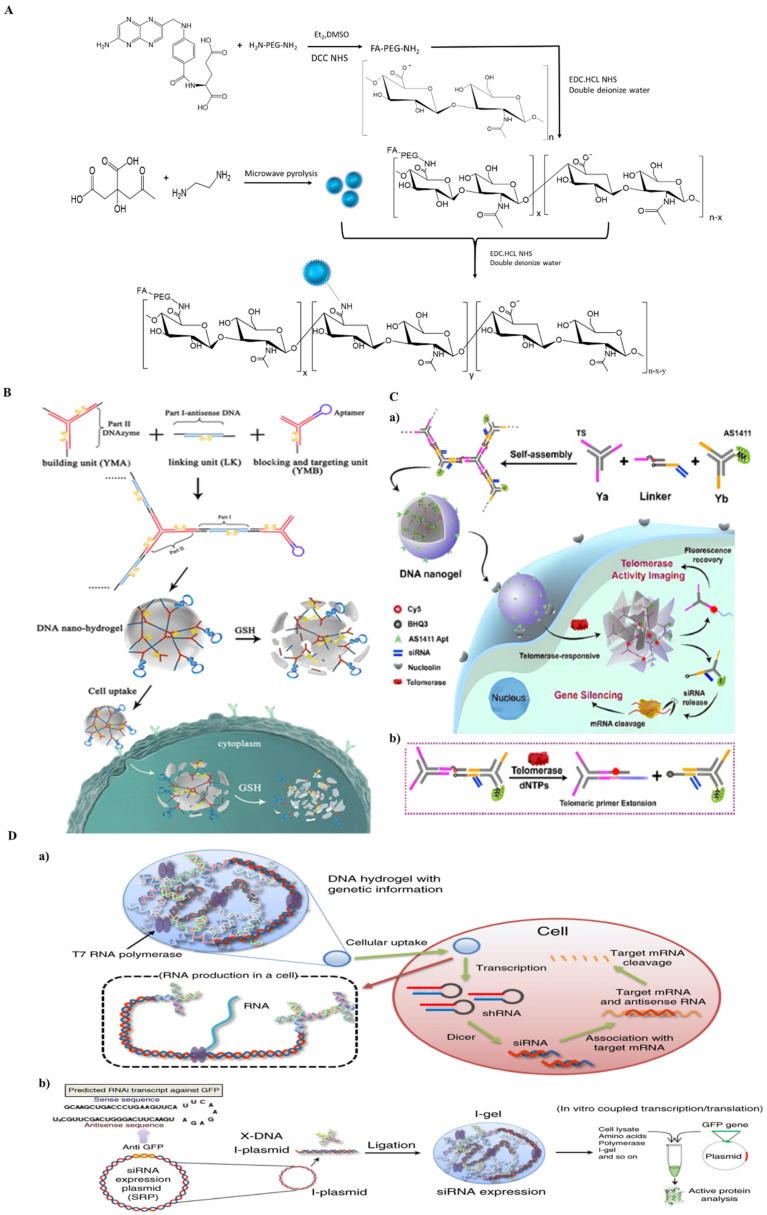
(**A**) Schematic illustration of the formation of PEGHA@CD hybrid nanogels. (Reproduced with permission from ref. [89] Copyright Elsevier 2016.) (**B**) Schematic illustration of stimuli-responsive DNA nanohydrogel formation scheme. The Y-shaped monomers (YMA and YMB) and linkers are designed to crosslink by hybridization of their “sticky end” segments (black lines), leading to nanohydrogel formation. (Reproduced with permission from ref. [90] Copyright © 2022, American chemical society) (**C**) (**a**) Illustration of the DNA nanogel for telomerase imaging and telomerase-activated gene therapy. (**b**) Telomerase-triggered telomeric primer extension for the recovery of the fluorescent signal and the release of siRNA. (Reproduced with permission from ref. [91] Copyright © 2022, American chemical society) (**D**) Illustration of RNAi mechanism of I-gel. (**a**) A schematic diagram illustrating the I-gel mechanism in a living cell. (**b**) A schematic diagram illustrating the I-gel mechanism in a cell lysate assay. (Reproduced from ref. [92] with permission of Nature Communication 2018).

Hyaluronic acid is an anionic polysaccharide composed of D-glucuronic acid and N-acetyl-D-glucosamine. Hyaluronic-based nanohydrogel has shown great potential for nanotherapeutics and nanomedicine because of its unique properties. Hyaluronic-based nanohydrogels have been widely employed in drug delivery processes [86,87]. Luan et al. reported the fabrication of pH responsive hyaluronic-based nanohydrogel for cancer specific target drug delivery. Hyaluronic-based nanohydrogels were synthesized using the surfactant free one-pot method by ketal crosslinkers. DMAEP (2,2-dimethacroyloxy-1-ethoxypropane) as a crosslinker and radical polymerization mechanism was enacted to obtain hyaluronic-based pH responsive nanohydrogels. These biodegradable and significantly constructive nanohydrogels were employed to deliver doxorubicin drugs using pH responsive drug release behavior [88]. Jia et al. have earlier reported the nanohydrogels based on hyaluronic acid, which are crosslinked with carbon dots as dual receptor-mediated targeting tumors with theranostic features. (Figure 4A) The study reports that the applied use of hyaluronic acid nanohydrogels can be used for real time and noninvasive location tracking to cancer cells. Jia et al. have shown, for the first time, folate-terminated poly(ethylene glycol) modified hyaluronic acid (FA-PEG-HA) with carbon dots via crosslinking. Doxorubicin-loaded hyaluronic nanohydrogel possesses an ideal release in a weak acid environment, while in neutral media it does not show any release, which demonstrates the pH sensitive behaviour of nanohydrogels. Hyaluronic nanohydrogels have been widely studied due to their compatibility with other nanomaterials and their non-cytotoxic nature [89].

#### 3.1.9. DNA-Based Nanohydrogel

DNA nanohydrogels have received considerable attention as an alternative to polymeric materials [93]. Especially earlier reported studies of DNA hydrogels have shown the versatile role of DNA hydrogels in biomaterials and active biomedical applications [94]. Firstly, DNA-based hydrogels have been classified based on crosslinking and self-assembly of DNA based strands and motifs [95,96]. Hybrid DNA hydrogels were synthesized by the coupling and crosslinking of natural or synthetic polymers or biomolecules attached to DNA scaffolds [97,98,99]. DNA hydrogels are comprised of (3D) three-dimensional networks of polymeric chains. Park et al. have previously developed DNA based materials with wide applications in such things as multiplexed diagnosis materials and vaccines in drug delivery, and in cell free protein production [100]. Similarly, Cheng et al. reported fast pH responsive DNA hydrogel assembly by the formation of intramolecular i-motif structures that can be switched from a gel to a non-gel state [101]. DNA-based hydrogels have attracted considerable attention as in diagnostics [102] and plasmonics [103]. Recently, numerous studies have been developed to overcome difficulties relevant to serving nanotherapeutics using nanohydrogels [104]. Hur et al. have studied DNA hydrogel and engineering biomaterials to successfully contribute to broadening the area of nanotherapeutics using DNA as a nano hydrogelator [105]. Song et al. have reported several studies on using DNA nanohydrogel as a delivery vehicle for gold nanorods and doxorubicin as a photothermal and a chemotherapeutic [103]. The two photothermal and chemo cancer agents were assembled via electrostatic interactions and DNA strand ligations, respectively. Light-triggered and highly synergistic combination cancer therapy was demonstrated in cellular and animal models [106]. In cancerous cells, glutathione can play both protective and pathogenic roles; glutathione is a substance made from the amino acids glycine, cysteine, and glutamic acid. It is produced by the liver and has been involved in many body processes. Glutathione is involved in tissue building and repair, making chemicals and proteins needed in the body and for immune system functions. Li et al. have reported the self-assembly of DNA nanohydrogels with stimuli-responsive properties in targeting and regulating gene therapy. Here, they report DNA nanohydrogel formation using Y-shaped monomers and linkers which were designed to crosslink by hybridization of their sticky end segments leading to nanohydrogel formation. The self-assembly of Y-shaped DNA shows easy synthesis without enzymatic ligation or photopolymerization, with controlled size, efficient cellular uptake, and enhanced nuclease resistance. The study shows the incorporation of different functional elements, such as disulfide linkages, and therapeutic genes into different molecular building blocks. The synthesis of cancer cell-specific aptamer-based nanohydrogels could be used for targeted gene therapy using stimuli-responsive behaviour (Figure 4B) [90]. Lin et al. developed an intelligent DNA nanohydrogel with specific targeting capability that can be stimuli activated for both in vitro telomerase detection and in vivo telomerase-triggered gene therapy (Figure 4C) [91]. Gene therapy and protein synthesis has been a critical approach with restrictive methodologies. Protein production mainly reports extraction from animals or expression with engineered bacteria. Protein production in cell-free medium to gene silencing and gene stability, has been widely studied. Song et al. have reported on an RNA producing DNA nanohydrogel that is able to produce RNA in a cell and enhances the efficient RNA interference mechanism to silence target genes. This RNAi exhibiting gel consists of a plasmid that carries the gene transcribing siRNA against the target mRNA as part of the gel scaffold. The RNAi efficiency of the synthesized gel has been confirmed by green fluorescent protein (GFP) expression assay and RNA production quantification (Figure 4D) [92].

### 3.2. Synthetic Polymer Based Nanohydrogels

A synthetic polymer is a class of polymer encompassing all the materials constituted of long molecular chains and organic connections obtained through the processing of natural products or the synthesis of primary materials from oil, gas, etc. Synthetic polymer hydrogels are designed to provide an alternative with further advantageous polymeric networks. Synthetic polymers are known for their controlled structure and desired mechanical properties, although they do not have any inherent bioactivity. Synthetic polymers classified as hydrogels are classified as either homopolymers, copolymers, or interpenetrating networks. (Figure 2) Homopolymers and copolymers contain only one type of polymer. Homopolymers contain only one type of monomer, whereas copolymers contain two or more types of monomers in their chains. Interpenetrating networks consist of two different types of polymer chains that are crosslinked with each other. The interpenetrating networks are synthesized using the simultaneous polymerization of two different types of precursor polymers produced through independent synthetic routes. However, a sequential interpenetrating network involves simultaneous interpenetrating networks and the polymerization of the second type of monomer inside a swollen single polymer-based network. Synthetic polymer based nanohydrogels were further functionalized to be utilized in a multifunctional role. Synthetic polymers are the other known nano hydrogelators that are abundantly used in the construction of nanohydrogels with different biomaterials. Synthetic polymers are less immunogenic and do not cause chronic immunogenic inflammation. Synthetic polymers are also known to have better mechanical properties when compared to natural polymers. The biodegradability of the synthetic polymers can also be adjusted, and this makes them suitable for tissue engineering applications. Several synthetic polymers such as polylactic acid (PLA), polyvinyl alcohol (PVA), polylactic-co-glycolic acid (PLGA), poly (l-lactic acid) (PLLA), and polydioxanone (PDO) are the most studied due to their potency in biomedical applications. (Figure 2) The development of nanohydrogels using synthetic polymers has been evolved in past decades [107]. Few studies have reported the Poly(ethylene glycol) based nanohydrogels for drug delivery, Poly(ethylene glycol) is a non-degradable polymer, though it could be functional with the modifications with degradable moieties [108]. Modified surfaces of the nanohydrogels/drug delivering vehicles/carriers offer an alternative way to modulate therapeutic outcomes in tumor sites with the precise target via targeting ligands [109,110]. Moreover, synthetic, natural, and hybrid polymers have been vastly utilized for nanodevice roles [111,112]. As earlier discussed, attachments of non-degradable polymers with degradable polymers with nanomaterials are increasing concerns in present research [113,114]. The copolymer networks of different types of polymers that increase the biodistribution and contribute to the therapeutics according to their active roles. Zhou et al. have studied the role of oxidized alginate crosslinking of the PEGylated oxidized alginate (mPEG-OAL) with cyclodextrins (α-CD). In this study, the author claims that for the first time, controlled release performance of pH and reduction dual responsive oxidized alginate doxorubicin (mPEG-OAL-DOX/Cys) prodrug nanohydrogels were investigated [115]. The concept of the copolymer and interpenetrating polymers are leading concepts which help to reveal the versatile role of polymeric networks.

## 4. Functionalized Role of Nanohydrogels

**Figure 5:**
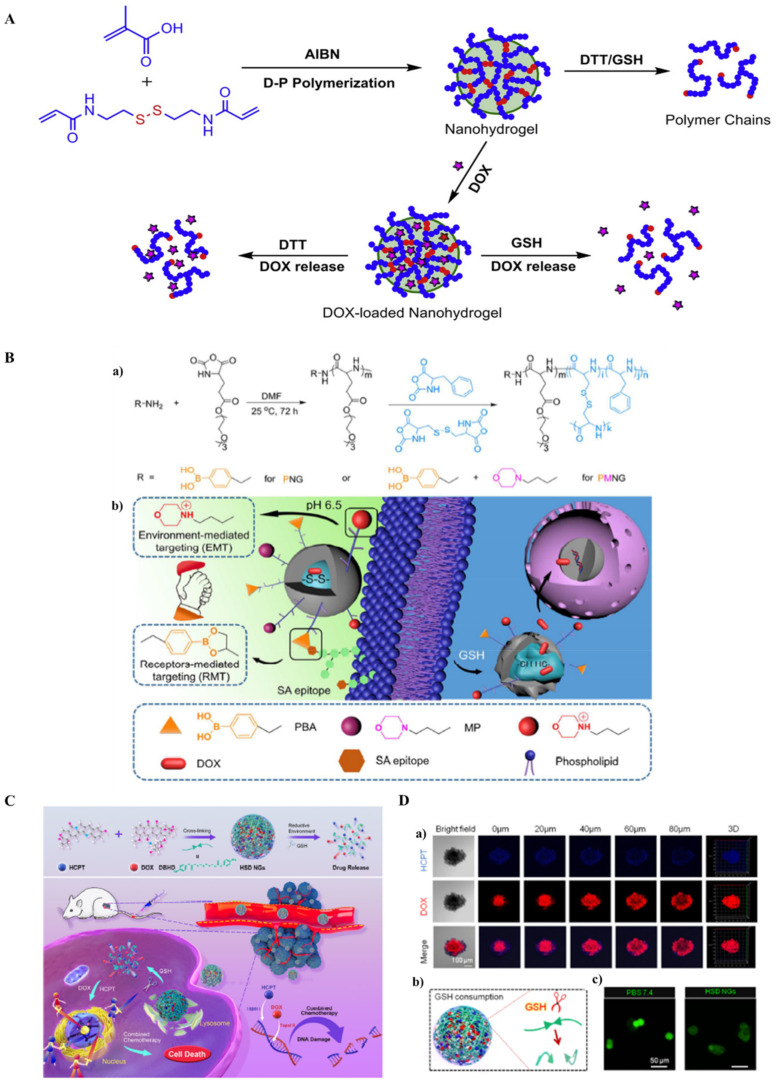
(**A**) Illustration of the preparation, biodegradable behavior, and redox stimuli-responsive drug release of the PMAA nanohydrogels. (Reproduced with permission from ref. [120] Copyright Elsevier 2012) (**B**) Schematic representation of (**a**) Synthesis route of nanohydrogels. (**b**) Targeting mechanism of phenylboronic acid and morpholine nanohydrogels. (Reprinted with permission from ref. [121] Copyright @American chemical society 2017) (**C**) Schematic Illustration of reduction-responsive chemo-capsule-based prodrug nanohydrogel for enhancing the treatment of chemotherapy (**D**) Deep tumor tissue penetration and GSH response of HSD nanohydrogels. (**a**) Confocal images of MCSs at different depths that were treated with HSD nanohydrogels for 6 h. (**b**) Illustration of the disulfide bond breakage in response to GSH. (**c**) Fluorescence images and fluorescence quantitative results of GSH consumption of 4T1 cells after being treated with HSD nanohydrogels for 6 h. (Reproduced with the permission from ref. [122] Copyright @American chemical society 2021).

The controlled delivery of drugs and the robust functionalization of nanohydrogels demands a biologically adaptable driving force to perform the active roles. Stimuli responses are the process that resembles the mechanical activity of biomolecular components. The mechanism of the extracellular matrix and biomolecules inside the cells involves the pH/redox/glutathione/glucose/lysosomal enzymes functionalization. Similarly, external stimuli-response involves temperature/magnetic field/ultrasound/and light [44,72,116,117,118,119]. Drug delivery, nucleic acid delivery, and polypeptide delivery require spatial control and temporal control, which are necessary in order to achieve optimal therapeutic efficacy [29]. Researchers have been keen to use such mechanisms to deliver the drugs and perform the non-external force incorporated activities which reduce the side effects to a negligible level. Pan et al. earlier reported dual stimuli-responsive biodegradable nanohydrogels. Poly(methacrylic acid) based nanohydrogel was prepared from methacrylic acid and an N,N-bis(acryloyl)cystamine crosslinker by distillation precipitation polymerization. The nanohydrogels were loaded with doxorubicin with a strong electrostatic interaction between amine and carboxyl groups from nanohydrogels at a physiological pH (Figure 5A) [120]. Chemotherapeutics studies have been widely studied with the incorporation of stimuli-responsive elements. Chemotherapy has shown several advantages. On the contrary, restricted therapeutic effects and unwanted drug resistance effects are the major clinical hurdles. Chen et al. reported targeted chemotherapeutics for metastatic malignancy. The author has studied and described the effective targeting chemotherapeutics, which can obliterate the off-target effects and reduce tumor homogeneity. Owing to receptor mediated targeting and environment mediated targeting, Chen et al. have developed a dual targeting nanohydrogel with phenylboronic acid and morpholine. A combined phenylboronic acid ligand in nanohydrogel selectively binds to the overexpressed sialyl antigen in highly metastatic tumor cells, whereas the morpholine ligand targets the extracellular pH condition, which helps with cell internalization. Moreover, the reductive agent glutathione incorporates in the reductive release of chemotherapeutics (Figure 5B) [121]. Similarly, Lu et al. reported the prodrug nanohydrogel in response to the reductive tumor microenvironment, with high tissue permeability and tumor accumulation. The authors have shown in the studies that nanohydrogels specifically enhance tumor growth inhibition in in vitro and in vivo by the synergistic DNA damage and the release of chemotherapeutic combined drugs by the reductive response. The study shows covalent conjugation of the hydroxyl group, which contains topoisomerase I and topoisomerase II inhibitors that are 10-hydroxy camptothecin and doxorubicin. The reductive agent glutathione was the factor responsible for releasing the drug effectively (Figure 5C,D). The studies and results show that the combined effects of 10-hydroxy camptothecin (HCPT) and doxorubicin (DOX) contribute to the apoptosis process through the topoisomerase pathway, which improves the inhibitory effect in tumor growth [122].

In recent times, clean water and environmental conservation have become the most prevalent global problems. These subjects are affecting humankind around the world, and therefore the interest of the scientific community in highlighting and modifying the potential application of hydrogel is similarly engaged [123]. The early reported work has attracted attention because of the fact that hydrogels can increase the water retention capacity of soil. Wastewater management uses metal ions to capture hybrid nanohydrogels. In addition, nanohydrogels can be employed in water treatments with a circular approach and tackle the conundrum associated with environmental toxins. Nanohydrogels could be a great alternative, with biocompatible and less harmful applications in agriculture, efficient fruit pest management, dye pollutant removal, and food grade Pickering emulsions [124,125,126]. Vundavali et al. have reported that they developed silver-coated nano clay composite cross linked polyacrylamide polymers. The synthesis was carried out by a polymerization reaction with acrylic acid, acrylamide, ammonium persulphate as initiator and N,N-methyl biscrylate as a cross-linker loaded with clay in the presence of nitrogen gas while the temperature was maintained at 65 °C [127]. The authors report that hydrogel has excellent water retention capacity that can be utilized in rainfed agriculture. Moreover, nanohydrogels have been employed in agriculture aspects for nutrient enrichment. Meurer et al. have reported on using nanohydrogels as a foliar fertilizer delivery system. The author synthesized nanohydrogels using inverse mini-emulsion polymerization methods (water in oil). Polyallylamine hydrochloride and N,N-methylene-bis(acrylamide) were dissolved in an aqueous phase and further dispersed in toluene containing the surfactant Span 80. The crosslinking of PAH was achieved by the Aza-Michael addition mechanism. They also manufactured nanohydrogels with controlled crosslinking density and pH responsive properties which can be utilized in sustainable agriculture [128]. Similarly, Pickering emulsion’s role in the majority of fields is well known, whereas nanohydrogel-mediated Pickering emulsion manufacturing has also been explored extensively. The usage of Pickering emulsion in oil and gas fields and its robust utilization in the petroleum industry is widely known [129,130,131]. Also, studies have suggested that the utilization of nanohydrogels could be an alternative to overcome limited crude oil recovery [132].

## 5. Conclusions and Outlook

The polymeric networks in strategic design and the classification of natural and synthetic polymers with reported nanohydrogels synthesis studies and applications of nanohydrogels are summarized from a polymeric perspective in this review. The programmable structures, physiochemical and nanotherapeutic properties of nanohydrogels are discussed. Notably, nanohydrogels exhibit a series of unique properties based on their monomer/polymer building types and sources. Nanohydrogel construction has proven to be a desirable research platform for applied applications of polymeric molecules in the nanoscale. Nanomaterials have been extensively performing well; however, nanohydrogels have shown superior properties over nanomaterials due to their flexibility, softness, and better pharmacokinetics. The resemblance of nanohydrogels to tissues and their feasible deformation and formation makes them more efficient in overcoming biological barriers. Similarly, several nanohydrogels could modify peptides and other targeting moieties for onsite delivery. As earlier reported earlier, hybrid nanohydrogels are the beneficial alternative. The emerging hybrid nanohydrogels are being produced and studied widely based on their applications. Several earlier reports have summarized detailed studies on hybrid nanohydrogels used with carbon-based nanohydrogels. Hybrid/synthetic/natural nanohydrogels have shown their biomedical applications abundantly, whereas synthetic polymers have been effectively employed for environmental purposes. However, the challenges of polymers, such as their polymer-based properties, restrict nanohydrogel performance. As reported earlier, studies have shown that the changes in the properties of polymers are due to the presence of external medieties and attachments. Nanohydrogels have been employed diversely, and they could provide a possible alternative in nanotechnology to overcome cytotoxic effects. Nanohydrogels can be well equipped with the desired features of optics, electronics, and other physical properties. Nanohydrogels with enhanced properties such as high sensitivity, ultrafast sensing, and soft robotics for drug release at the clinical phases are yet to be realized. Biomedical and non-biomedical applications are still the major concerns that need to be resolved. We anticipate that this review can help researchers to gain a better understanding of the classification-based evolution of nanohydrogels and their robust functionalization.
